# Supplementary material for: Peripancreatic Adipose Tissue Remodeling and Inflammation during High Fat Intake of Palm Oils or Lard in Rats
Source: Nutrients. 2021 Mar 30;13(4):1134. doi: 10.3390/nu13041134 (PMC8065769; doi:10.3390/nu13041134)
Supplement: Supplementary file 1 [file nutrients-13-01134-s001.pdf]

Supplementary Materials

Supplementary Table S1 Fatty acid composition of oils and lard used in diets

FATTY ACIDS	**Control**[a] % mol/mol	**crude PO**[b] % mol/mol	**refined PO**[b] % mol/mol	**Lard**[b] % mol/mol
C14	0	0.5	0.5	1.3
C16	12.0	38.1	36.5	24.9
16:1n-7	0.6	0.1	0.2	2.8
C18	3.8	5.9	4.9	15.5
18:1n-9	19.7	43.2	46.4	43.4
18:1n-7	0	0.6	0.6	0
18:2n-6	55.4	10.0	9.6	9.5
18:3n-3	8.0	0.3	0.2	0.8
C20	0.5	0.4	0.3	1.3
20:1n-9	0	0.1	0.2	0.9
20:1n-7	0	0.2	0.2	0
Saturated	16.3	45.1	42.4	43.4
Monounsaturated	20.3	44.4	47.7	47.1
Polyunsaturated	63.5	10.5	9.9	9.6

crude PO: crude palm oil; refined PO: refined palm oil.

[a]: Composition of soybean oil according to *Souci et al*. [1]
[b]: Data obtained in our laboratory by analyzing crude palm oil, refined palm oil and lard used in this study.

Supplementary Table S2 Comparison of Antioxidant content in crude PO and refined PO

		Crude PO	Refined PO	Refined PO / Crude PO (%)
	**Kato et al. (1981)** [2]			
Vitamin E	α-Tocopherol (μg/g)	194	171	88.1%
	α-Tocotrienol (μg/g)	176	145	82.4%
	γ-Tocotrienol (μg/g)	333	222	66.7%
	δ-Tocotrienol (μg/g)	93	46	49.5%
	Total Vitamin E (μg/g)	796	584	73.4%
	**Wong et al. (1988)** [3]			
	Total Vitamin E (μg/g)	794	643	81.0%
	**Nagendran et al. (2000)** [4]			
	α-Tocopherol (μg/g)	187	139	74.3%
	α-Tocotrienol (μg/g)	208	163	78.4%
	γ-Tocotrienol (μg/g)	376	205	54.5%
	δ-Tocotrienol (μg/g)	98	54	55.1%
	Total Vitamin E (μg/g)	869	561	64.6%
	**Szydłowska-Czerniak et al. (2011)** [5]			
Antioxidant capacity	FRAP Method (μmol TE/100 g)	128.7	25.6	19.9%
	DPPH Method (μmol TE/100 g)	127.5	25.4	19.9%
Polyphenols	Total Phenolic content (mg GAE/100 g)	6.1	4.5	73.8%
Carotenoids	Total Carotenoid Content (mg β-carotene E/100 g)	43.8	0.3	0.7%

Published data regarding antioxidant capacity, amounts of vitamin E, carotenoids and polyphenols in palm oil (PO) before and after the refining process. Data presented relate to classical physical refining of PO.
Antioxidant capacity have been assessed by ferric ion reducing antioxidant power (FRAP) and 2,2-diphenyl-1-picrylhydrazyl (DPPH) methods and expressed as trolox (6-hydroxy-2,5,7,8-tetramethylchroman-2-carboxylic acid) equivalent (TE).
Total phenolic content is expressed as gallic acid equivalent (GAE) and carotenoid content as β-carotene equivalent (β-carotene E).

1. Souci, S.W.; Fachmann, W.; Kraut, H. *Food Composition and Nutrition Tables*; 6th ed.; Medpharm GmbH Scientific: Stuttgart, Germany, 2000;
2. Kato, A.; Gapor, A.; Tanabe, K.; Yamaoka, M.; Mamuro, H. Esterified α-Tocopherol and Tocotrienols in Palm Oils. *Journal of Japan Oil Chemists' Society* **1981**, *30*, 590–591, doi:10.5650/jos1956.30.590.
3. Wong, M.L.; Timms, R.E.; Goh, E.M. Colorimetric Determination of Total Tocopherols in Palm Oil, Olein and Stearin. *J Am Oil Chem Soc* **1988**, *65*, 258, doi:10.1007/BF02636412.
4. Nagendran, B.; Unnithan, U.R.; Choo, Y.M.; Sundram, K. Characteristics of Red Palm Oil, a Carotene- and Vitamin E–Rich Refined Oil for Food Uses. *Food Nutr Bull* **2000**, *21*, 189–194, doi:10.1177/156482650002100213.
5. Szydłowska-Czerniak, A.; Trokowski, K.; Karlovits, G.; Szłyk, E. Effect of Refining Processes on Antioxidant Capacity, Total Contents of Phenolics and Carotenoids in Palm Oils. *Food Chem* **2011**, *129*, 1187–1192, doi:10.1016/j.foodchem.2011.05.101.
